# The combined effect of a novel formula of herbal extracts on bacterial infection and immune response in *Micropterus salmoides*

**DOI:** 10.3389/fmicb.2023.1185234

**Published:** 2023-06-02

**Authors:** Huanyu Guo, Jing Chen, Xuemei Yuan, Jian Zhang, Jiayang Wang, Jiayun Yao, Haixia Ge

**Affiliations:** ^1^College of Life Sciences, Huzhou University, Huzhou, Zhejiang, China; ^2^Key Laboratory of Healthy Freshwater Aquaculture, Ministry of Agriculture and Rural Affairs, Key Laboratory of Fish Health and Nutrition of Zhejiang Province, Zhejiang Institute of Freshwater Fisheries, Huzhou, Zhejiang, China; ^3^State Key Laboratory of Natural Medicines, China Pharmaceutical University, Nanjing, China

**Keywords:** novel herbal combination, antibacterial activity, *Aeromonas hydrophila*, immune response, *Micropterus salmoides*

## Abstract

Herbal extracts have been considered as ideal alternative to antibiotics in aquaculture and application of combinatory effective extracts always can exhibit the enhanced bioactivity with high efficiency. In our study, a novel herbal extract combination GF-7, which is composed of *Galla Chinensis*, *Mangosteen Shell* extracts as well as the effective parts of *Pomegranate peel* and *Scutellaria baicalensis* Georgi extracts, was prepared and applied for the therapy of bacterial infection in aquaculture. The HPLC analysis of GF-7 was also investigated for quality control and chemical identification. In the bioassay, GF-7 had excellent antibacterial activity against various aquatic pathogenic bacteria *in vitro*, and the related MIC values were between 0.045 and 0.36 mg/mL. After feeding *Micropterus salmoide* with GF-7 (0.1, 0.3, and 0.6%, respectively) for 28 days, the activities of ACP, AKP, LZM, SOD, and CAT of the liver in each treatment group were significantly increased and the content of MDA was significantly decreased. Meanwhile, the hepatic expression of the immune regulators including *IL-1β*, *TNF-α*, and *Myd88* at different times was up-regulated in varying degrees. The challenge results exhibited a good dose-dependent protective effect on *M. salmoides* infected with *A. hydrophila*, which was further confirmed by liver histopathology. Our results imply that the novel combination GF-7 is a potential natural medicine for the prevention and treatment of numerous aquatic pathogenic infectious diseases in aquaculture.

## 1. Introduction

Aquaculture has significantly advanced in recent years to provide nutrient-dense and healthful protein sources for humans (Abdel-Latif et al., 2023), in which fish consumption account for a large percentage and is anticipated to reach two-thirds of all aquaculture globally by 2030 (Beltran and Esteban, 2022). Due to the increasing requirement for aquatic fish, the level of large-scale and intensive farming caused the issue of aquatic animal disease to become serious. Bacterial infectious disease is one of the related aquatic animal diseases that impede the sustainable and healthy development of aquaculture, resulting in financial losses for the sector (Li et al., 2021b). A previous study has shown that the main aquatic pathogenic bacteria include *Aeromonas*, *Vibrio*, *Streptococcus*, *Edwardsiella*, *Nocardia* and so on (Du et al., 2022). Particularly, when aquatic animals are infected with *Aeromonas* species, significant morbidity and mortality can rate up to 100% (Dien et al., 2022). Among them, *Aeromonas hydrophila* can infect most farmed fish, and cause diseases such as bacterial enteritis, bacterial septicemia, motile Aeromonas septicemia (MAS) and so on (Ibrahim et al., 2022; Jin et al., 2023), resulting in mass deaths and economic losses to the aquaculture industry (Li et al., 2021a).

Antimicrobial medications supplemented in feeds are extensively used for the prevention and treatment of bacterial fish disease (Cabello, 2006). However, medication chemical supplementation over a long period caused their residue in the environment and antibiotic resistance (Cabello et al., 2016). Persistent antibiotic exposure to the environment even at quantities below the minimum inhibitory concentration, can exert selective pressure on the bacterial populations in multi-bacterial matrices including ponds, sediments, and biofilms, which contribute to the transfer of antimicrobial genes across bacteria (Santos and Ramos, 2018). More seriously, residues of antimicrobial or bacteria resistant to antimicrobials and resistance genes can be transferred from aquatic environments to terrestrial livestock and humans, which increases the risk of wide-spreading drug resistance (Santos and Ramos, 2018). Vaccination is considered as a potentially useful management approach in aquaculture as a prophylactic measure to protect fish. However, the official vaccinations against fish infectious are limited and their biological activities are unable to show continuous effects against infection to improve immature fish immune systems (Pridgeon and Klesius, 2012). Therefore, it is necessary to develop and evaluate other therapeutical approaches to address antimicrobial resistance (AMR).

Any chemical or management method that can substitute therapeutic medications that are becoming less effective against bacteria as a result that AMR is known as an alternative to antibiotics (ATAs). Chinese herbal medicine has been used commonly as a “green drug” to replace chemotherapeutics and antibiotics to prevent and treat fish diseases because of their good therapeutic effectiveness, small adverse effects, low toxicity, diverse drug targets, and less possibility to develop drug resistance (Reverter et al., 2014). The bioactive components from Chinese herbal remedies, such as polyphenols, alkaloids, flavonoids, coumarins, saponins, polysaccharides, anthraquinone, and terpenoids have been applied to control and prevent fish diseases (Zhang et al., 2022). Recent research indicated that the antibacterial effects of Chinese herbal medicine mainly focused on the direct destruction of pathogenic bacteria (Li et al., 2017; Fei Wang et al., 2020) and immunological stimulation (Xu et al., 2020; Ze-Hao Ding et al., 2021; Hou et al., 2022; Raissy et al., 2022), both of which participate in aquatic animals’ disease resistance.

*Scutellaria baicalensis* (*Scutellaria Radix,* SB) and *Pomegranate peel* (PoP) have been widely used in the field of aquaculture, with obvious immune enhancement effect and disease resistance (Xu et al., 2020; Hamed and Abdel-Tawwab, 2021; Ze-Hao Ding et al., 2021). Flavonoids isolated from SB and polyphenols extracted from PoP have been proven to be essential herbal extracts with effective antibacterial, antioxidant and immune protection (Zhao et al., 2019; Mo et al., 2022). Meanwhile, *Mangosteen Shell* (MS) and *Galla Chinensis* (GC) also exhibited good antibacterial activity against aquatic pathogenic microorganisms in fish (Meepagala and Schrader, 2018; Wu et al., 2021). It is worth for us to make a combination of these herbal plants and applied for bacterial infections in fish.

In our previous work, we evaluate the antibacterial activity of different effective parts from various herbal extracts, screened and made a novel combination GF-7 with synergistic effect through the dual activity screening *in vivo* and *in vitro* (data not showing). This paper aims to show the inhibitory effects of the GF-7 (including GC and MS extracts, the effective fractions of PoP and SB) on various aquatic pathogenic bacteria, assess the effects of GF-7 on the immune responses and disease resistance against *Aeromonas hydrophila* in *Micropterus salmoides.*

## 2. Materials and methods

### 2.1. Preparations and HPLC analysis of GF-7

The novel combination GF-7 was composed of GC extract, MS extract, the effective fraction of PoP, and the effective fraction of SB in equal proportions. The specific preparation method and determination of the representative components of four Chinese herbal medicine were shown in the Supplementary material.

The GF-7 was provided by thoroughly mixing all four extracts or effective fractions in equivalent weight. HPLC analysis was conducted on the Agilent HPLC series 1260 system. The mobile phase consisted of water with 0.5% formic acid (A) (Shanghai Macklin Biochemical Co., Ltd.) and methanol (B) (Shanghai Macklin Biochemical Co., Ltd.) using a gradient program as follows: 8–30% B (15 min), 30–50% B (20 min), 50–95% B (25 min), 95–95% B (5 min), 95–8% B (15 min), 8–8% B (5 min) at a flow of 1 mL/min and DAD detection at 254 nm. The temperature was held constant at 30°C and the total run time was 85 min. The standard baicalin (LOT: Z28S11X125952), baicalein (LOT: C06M11Y112461), wogonoside (LOT: A09GB144759), wogonin (LOT: T11J11R108209), γ-mangostin (LOT: P13M6S1), α-mangostin (LOT: A29GB159282) were purchased from Shanghai Yuanye Biotechnology limited company. Gallic acid (LOT: 19010307) and ellagic acid (LOT: BGZ930) were purchased from Shanghai Bide Pharmatech limited company and Changsha Chemico Biotechnology limited company, respectively.

### 2.2. *In vitro* determining antibacterial activity

#### 2.2.1. Pathogenic bacterial strains

There were four strains of *Aeromonas hydrophila* from different sources: TPS-30, provided by Zhejiang Institute of Freshwater Fisheries, was originally isolated from infected crucian carp; BYK19103 and BYK19134, provided by Shanghai Ocean University, was originally isolated from the infected *grass carp* and *Chinese velvet crab*, respectively; ATCC GIM1.551 was purchased from Guangdong Provincial Microbial Culture Collection Center (GPMCCC). There were two strains of *Edwardsiella* from different sources: *Edwardsiella tarda* ATCC15947 was purchased from GPMCCC; *Edwardsiella ictaluri* AW30726K provided by Zhejiang Institute of Freshwater Fisheries, was originally isolated from infected *Pelteobagrus fulvidraco*. *Aeromonas veronii* (TH0426) was originally isolated from the infected *Pelteobagrus fulvidraco*; *Streptococcus dysgalactiae* (CCTCC M2018843) was originally isolated from infected *Chinese Sturgeon*; *Streptococcus agalactiae* (HNLFYL4) was originally isolated from infected *tilapia*; *Vibrio mimicus* (Hsy0531-K) was originally isolated from infected *Pelteobagrus fulvidraco*; *Aromonas punctata f. intestinalis* (CDQ-1) was originally isolated from infected *grass carp*, were provided by Zhejiang Institute of Freshwater Fisheries.

#### 2.2.2. Minimum inhibitory concentration determination

According to the clinical and laboratory standards institute (CLSI) standard (CLSI, 2022), the minimum inhibitory concentration (MIC) was determined by the plate double dilution method. The bacteria strain was inoculated in Mueller-Hinton (MH) liquid culture medium (Hangzhou Microbial Reagent Co., Ltd.) and cultured in a constant temperature shaker (Taicang Hualida Experimental Equipment Co., Ltd.) at 28°C for 16 h, and the bacterial solution was diluted to 1 × 10^8^ CFU/mL with normal saline (standard plate counting method). Inoculated 2.5 μL/point of bacterial suspension onto MH agar medium (Hangzhou Microbial Reagent Co., Ltd.) containing different concentrations of new combination GF-7 (0.023–0.72 mg/mL), and set 3 replicates at each inoculation point. Then, the plates were placed upside down in a 28°C incubator (Sanyo Electric Co., Ltd., Japan) for 24 h and the results were observed. Enrofloxacin hydrochloride (Shanghai Aladdin Biochemical Technology Co., Ltd.) was used as the positive control and DMSO (Shanghai Aladdin Biochemical Technology Co., Ltd.) as the negative control. If no bacterial growth was observed by the naked eye, it is recorded as the MIC of the GF-7 against the bacterial pathogens.

### 2.3. Scanning electron microscope

To determine the morphological changes of *A. hydrophila* TPS-30 treated with the GF-7, SEM studies were carried out according to the reported method with some modifications (Zhang et al., 2016). Strain TPS-30 was cultured in fresh MH liquid medium to the logarithmic stage, treated with GF-7 with the final drug concentration of 2MIC, and incubated in a shaker at 28°C for 8 h. After centrifugation at 5000 rpm for 10 min at 4°C and washing with sterile PBS (Thermo Fisher Scientific Shier Technology Co., Ltd.) 3 times, the lower layer precipitation was collected and then fixed with 2.5% glutaraldehyde (Shanghai Aladdin Biochemical Technology Co., Ltd.) at 4°C for SEM (SU8100, Hitachi, Ltd., Japan) analysis.

### 2.4. Fish rearing and fish feed

*Micropterus salmoides* (weight about 20 ± 2 g) were purchased by a fish farm in Balidian Base of Zhejiang Institute of Freshwater Fisheries. The healthy *M. salmoides* were kept for 2 weeks to adapt to laboratory conditions.

The 360 healthy *M. salmoides* were randomly divided into four experimental groups: control group (0%), low-dose group (0.1%), medium-dose group (0.3%) and high-dose group (0.6%). Each group had three replicates, and each parallel group had 30 fish (90 fish per group). The fish was reared into 300 L tanks holding dechlorinated freshwater at a density of 30 fish in each tank, respectively. To maintain the rearing conditions in the whole experiment, 30% water in each tank was replaced with dechlorinated freshwater daily to remove the metabolic wastes and uneaten food. During the experiment, each tank was continuously aerated, and the parameters of temperature, pH, and dissolved oxygen were maintained at 28 ± 1°C, 7.6–7.8, and not less than 6.0 mg/L, respectively.

The basic diet (crude protein ≥53.0%, crude fat ≥5.0%, crude fiber ≤4.0%, crude ash ≤16.0%, total phosphorus ≥1.0%, calcium ≥1.0%, lysine ≥2.7%, and moisture ≤12.0%) was purchased from Fujian Tianma Feed Co. LTD. GF-7 was added to the basic diet with the proportions of 0, 0.1, 0.3, and 0.6% (w/w), and then compressed into pellet feed, which was considered as the control group, low-dose group, medium-dose group, and high-dose group, respectively. All fish were fed twice a day at 9:00 a.m. and 5:00 p.m. with corresponding feed. The amount of each feeding was 3% of the fish’s weight.

### 2.5. Sampling procedure

During the feeding period, the livers of fish were collected every 7 days to evaluate immune-related enzyme activity and gene expression. In addition, fish samples were taken before the challenge test for a total of 5 times at the time points of 0 d, 7 d, 14 d, 21 d, and 28 d. Three fish per replicate (*n* = 9 per group) were randomly selected each time. After being fully anesthetized with 80 ppm eugenol (Shanghai Aladdin Biochemical Technology Co., Ltd.), the liver tissues of fish were dissected and stored at −80°C before use.

### 2.6. Determination of immune-related enzymes activity

The liver tissues were homogenized with normal saline 1:9 (m: V) according to the commercial kit instructions and centrifuged at 4°C, 3000 r/min for 10 min. The supernatant was collected as the tissue homogenate to measure the activities of acid phosphatase (ACP), alkaline phosphatase (AKP), superoxide dismutase (SOD), catalase (CAT) and lysozyme (LZM), and the level of malondialdehyde (MDA) by commercial kits (Nanjing Jiancheng Bioengineering Institute, Nanjing, China. ACP, item No.: A060-2, AKP, item No.: A059-2, SOD, item No.: A001-3, CAT, item No.: A007-1-1, LZM, item No.: A050-1-1, MDA, item No.: A003-1). All enzyme assays were performed in triplicate.

### 2.7. Determination of immune-related genes expression

The gene expressions level of *IL-1β*, *TNF-α*, *Myd88* and *Caspase3* in the liver of *M. salmoides* were evaluated by real-time PCR. The total RNA was extracted from liver tissue by RNA Extraction Kit (Luoyang Yiyan Biotechnology limited company). The concentration of RNA was determined by NanoDrop 2000 (Thermo Scientific, United States). The cDNA was synthesized by using a PrimeScript^™^ RT Master Mix (Takara, Japan). Briefly, Oligo dT Primer and Random 6 mers were used to reverse transcribe 200 ng RNA in the presence of 5 × PrimeScript RT Master Mix, and RNase Free dH_2_O at 37°C for 15 min, following inactivation at 85°C for 5 s. Finally, the synthesized cDNA was stored at −80°C until use.

Real-time PCR for the target genes was performed using a THUNDERBIRD SYBR qPCR Mix (TOYOBO, Japan) and quantified on the LightCycler^®^ 96 Instrument (Roche Diagnostics GmbH, Switzerland) using the following program: 5 μL of THUNDERBIRD SYBR® qPCR Mix, 3 pmol of Forward and Reverse Primers, 0.5 μL of cDNA template and nuclease-free water to a final volume of 10 μL. Then using the following program: denaturation step at 95°C for 1 min, followed by 40 amplification cycles of 15 s denaturation at 95°C, 30 s annealing at 60°C and 20 s extension at 72°C, followed by a Melting Curve Analysis. Primers were designed on NCBI and synthesized by Jinweizhi Biotechnology limited company (Table 1). The relative levels of the related genes were calculated based on the equation of 2^−ΔΔCT^ (Livak and Schmittgen, 2001). *β-actin* was used as the reference gene. Three independent duplicates were conducted for each of the data.

**Table 1:** Real-time PCR primer sequences.

Gene	Primer sequence	Product size (bp)	Accession/source
*IL-1β*	F-CTGAGCGACCGCAGTAAGAA	145	XM_038733429.1
	R-TGTTGCTTTCACAGACGGGA		
*TNF-α*	F-CAGTGCAATGGCAGAACCAG	127	XM_038723994.1
	R-TTGACCCTGAAGGACGCTTG		
*Myd88*	F-GGAAACTTTCGGACATGGCG	109	XM_038728827.1
	R-GGGTTGAGATACAGCCCCAA		
*Caspase3*	F-GCTTCATTCGTCTGTGTTC	98	(Yin et al., 2019)
	R-CGAAAAAGTGATGTGAGGTA		
*β-actin*	F-CATCCTCCGTCTGGACTTGGCT	257	(Yuan et al., 2020)
	R-CCTCTGGGCACCTGAACCTCT		

IL-1β, interleukin-1β; TNF-α, tumor necrosis factor α; Myd88, myeloid differentiation primary response gene 88; Caspase 3, cysteine-aspartic proteases-3.

### 2.8. Bacterial challenge

After 28 days of administration and sampling, 12 remaining fish were taken from each group for the challenge test to evaluate the protective effect of the GF-7 against *A. hydrophila* TPS-30. Before a challenge test, fish were examined to be free from *A. hydrophila* TPS-30 infection. And then all groups of *M. salmoides* were anesthetized and 0.1 mL *A. hydrophila* TPS-30 with a concentration of 4.52 × 10^8^ CFU/mL were injected intraperitoneally. The survival rates of each parallel group were observed for 7 days and fish mortality and protection rates were calculated by the equitation. To confirm *A. hydrophila* as the cause of mortality, recently dead and moribund fish were harvested and aseptically examined for re-isolation and identification of the challenging strain from different tissue samples (liver, spleen, and kidney).


$$\begin{array}{l} \mathrm{protection rate}\;\left(\%\right)=\\ \;\frac{\mathrm{control group mortality rate}-\mathrm{test group mortality rate}}{\mathrm{control group mortality rate}}\times 100 \end{array}$$

A simple flow chart of the experimental design of protective effect of GF-7 on *M. salmoides* infected with *A. hydrophila* is shown in Supplementary Figure S2.

### 2.9. Histological analysis of liver tissue

According to the reported method (Wang et al., 2022) with some modifications, liver specimens of *M. salmoides* were corrected, including normal and different dose groups (0, 0.1, 03, 0.6%) after the challenge, and fixed with 4% tissue cell fixation solution (Beijing Suolaibao Technology Co., Ltd.) at 4°C for histopathological analysis.

### 2.10. Statistical analysis

All data were expressed as mean value ± standard error (SE). Significance levels were determined by one-way analysis of variance (ANOVA) with SPSS 25, and the multiple comparisons were determined by Duncan test. Variance was subjected to homogeneity tests and the statistical significance level was set at *p* < 0.05.

## 3. Results

### 3.1. Preparation data and HPLC analysis of standardized novel combination GF-7

The GF-7 was composed of GC extract, MS extract, effective part of PoP, and effective part of SB in equal proportions. The extraction rates of four Chinese herbal medicines were 65, 17, 41, and 33%, respectively. We determined the content of the main components of the extracts or active parts of each Chinese herbal medicine in the GF-7 by UV or HPLC method (see Supplementary material), as shown in Table 2.

**Table 2:** Preparation data of four Chinese herbal medicines in the GF-7.

Raw materials	Extraction solvent	Extraction rate	Effective part	Representative components	Content
GC	50% ethanol	65%	−	Tannins	72.0%
MS	70% ethanol	17%	−	α-mangostin	20.5%
PoP	50% ethanol	41%	+	Tannins	49.2%
SB	70% ethanol	33%	+	Total flavonoids	75.2%

“−”, Extract was used in the GF-7; “+”, Effective part was used in the GF-7; GC, Galla Chinensis; MS, Mangosteen Shell; PoP, Pomegranate peel; SB, *Scutellaria baicalensis*.

**Figure 1:**
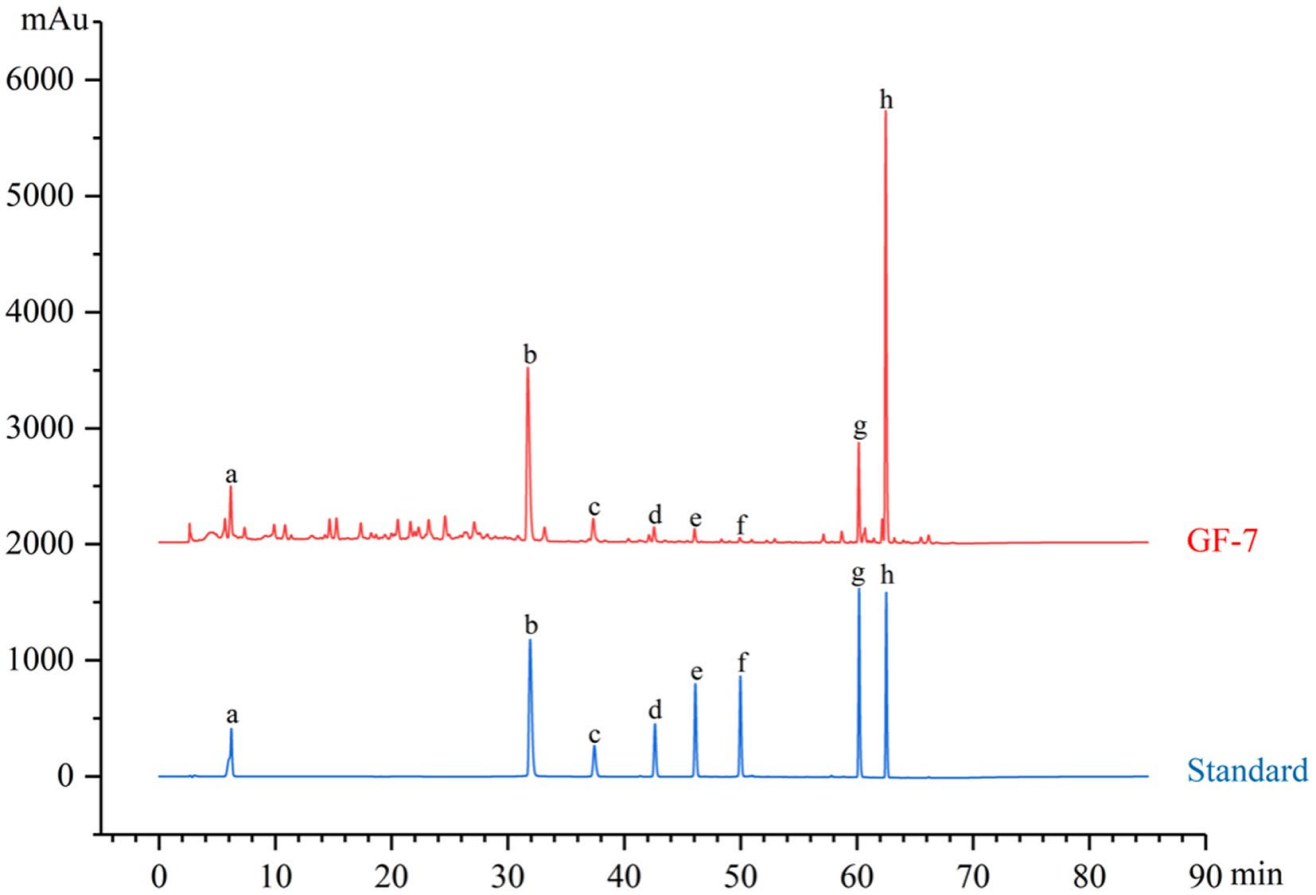
The HPLC fingerprint of GF-7. Standards: gallic acid (a), ellagic acid (b), baicalin (c), wogonoside (d), baicalein (e), wogonin (f), γ-mangostin (g), and α-mangostin (h).

In order to ensure the quality and stability of a complex system, it is necessary to perform a chemical composition analysis of GF-7. A typical HPLC analysis at an absorbance of 254 nm was developed for the chemical identification of GF-7. The ellagic acid from PoP, α- mangostin and γ-mangostin from the MS extract, baicalein, wogonoside, baicalein and wogonin from the effective fraction of SB, and gallic acid from GC extract had been identified as shown in Figure 1.

### 3.2. *In vitro* antibacterial activity of GF-7

**Figure 2:**
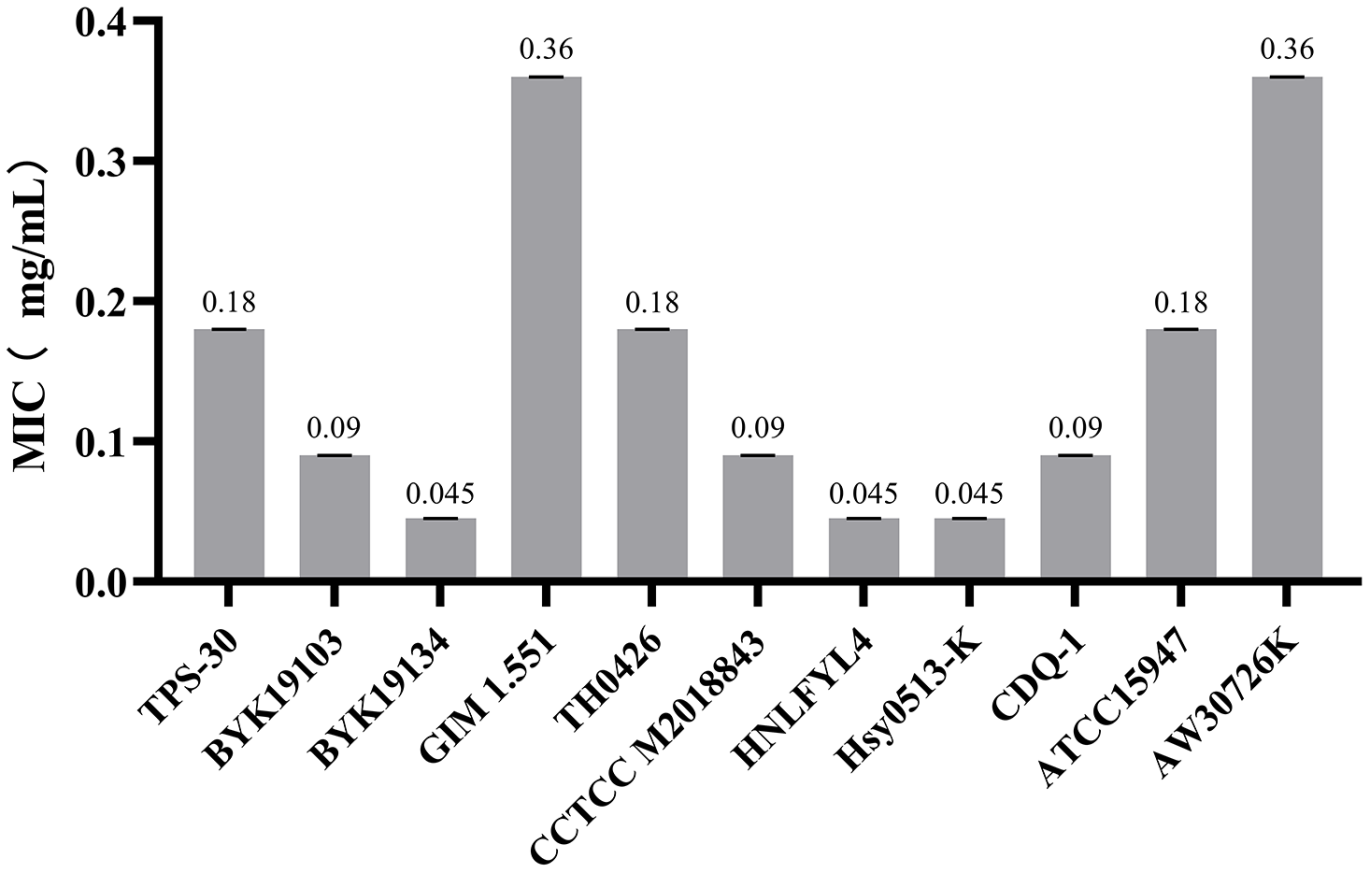
MICs of GF-7 on different pathogen strains.

In Figure 2, the GF-7 had a strong antibacterial effect on 7 species and 11 strains of aquatic pathogenic bacteria, indicating its inhibition on the pathogenic bacteria with a broad spectrum. Moreover, the inhibitory activity of four strains of *A. hydrophila* (TPS-30, BYK19103, BYK19134, GIM1.551) showed differences, and their MIC values ranged from 0.045 to 0.36 mg/mL; The inhibitory activities of two strains of *Edwardsiella* (ATCC15947, AW30726K) were varied within wide limits, and their MIC values were ranged from 0.18 to 0.36 mg/mL; Differently, GF-7 had a strong inhibitory effect on the growth of *A. veronii* (TH0426), *S. dysgalactiae* (CCTCC M2018843), *S. agalactiae* (HNLFYL4), *V. mimicus* (Hsy0513-K) and *A. punctata f. intestinalis* (CDQ-1), and their MIC values were ranged from 0.045 to 0.18 mg/mL.

### 3.3. Effects of GF-7 on the morphology and structure of *Aeromonas hydrophila* TPS-30

**Figure 3:**
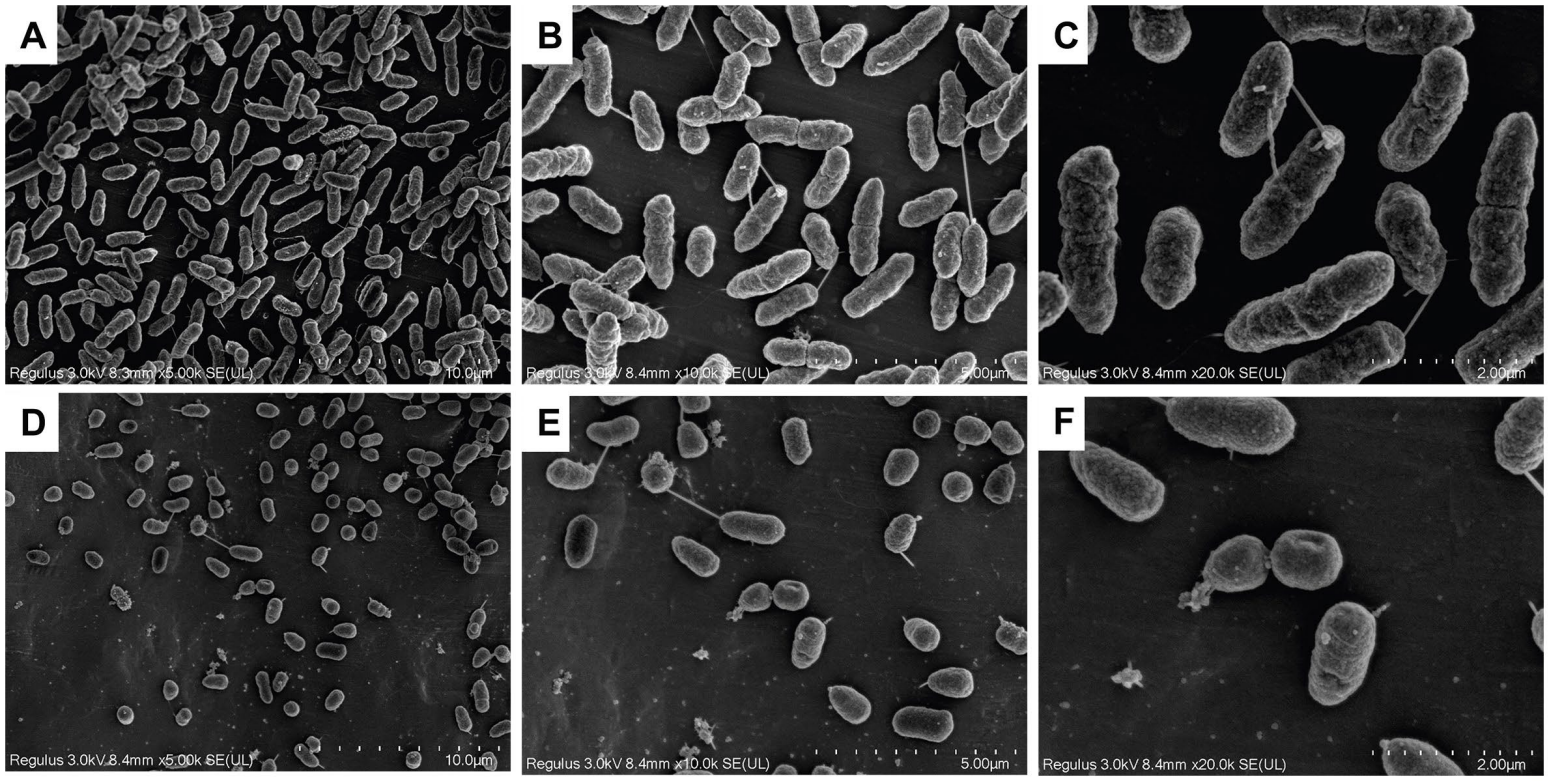
The scanning electron microscope (SEM) photography of TPS-30 with GF-7 treatment. **(A–C)** was the negative control group. **(D–F)** was the 2MIC-treated group. The multiples of **(A–C)** and **(D–F)** were 5,000, 10,000, 20,000, 5,000, 10,000 and 20,000, respectively.

GF-7 treatment could induce morphological changes of *A. hydrophila* as shown in Figure 3. GF-7 treatment could modify the surfaces of *A. hydrophila*, and significantly decrease the number of *A. hydrophila*. The normal cells were rod-shaped, regular and intact (Figures 3A–C), whereas the cells in GF-7 treatment groups became deformed, pitted and shriveled, and the damaged cell (Figures 3D–F) might result in the leakage of the cellular contents.

### 3.4. Effects of GF-7 on immune-related enzymes of *Micropterus salmoides*

**Figure 4:**
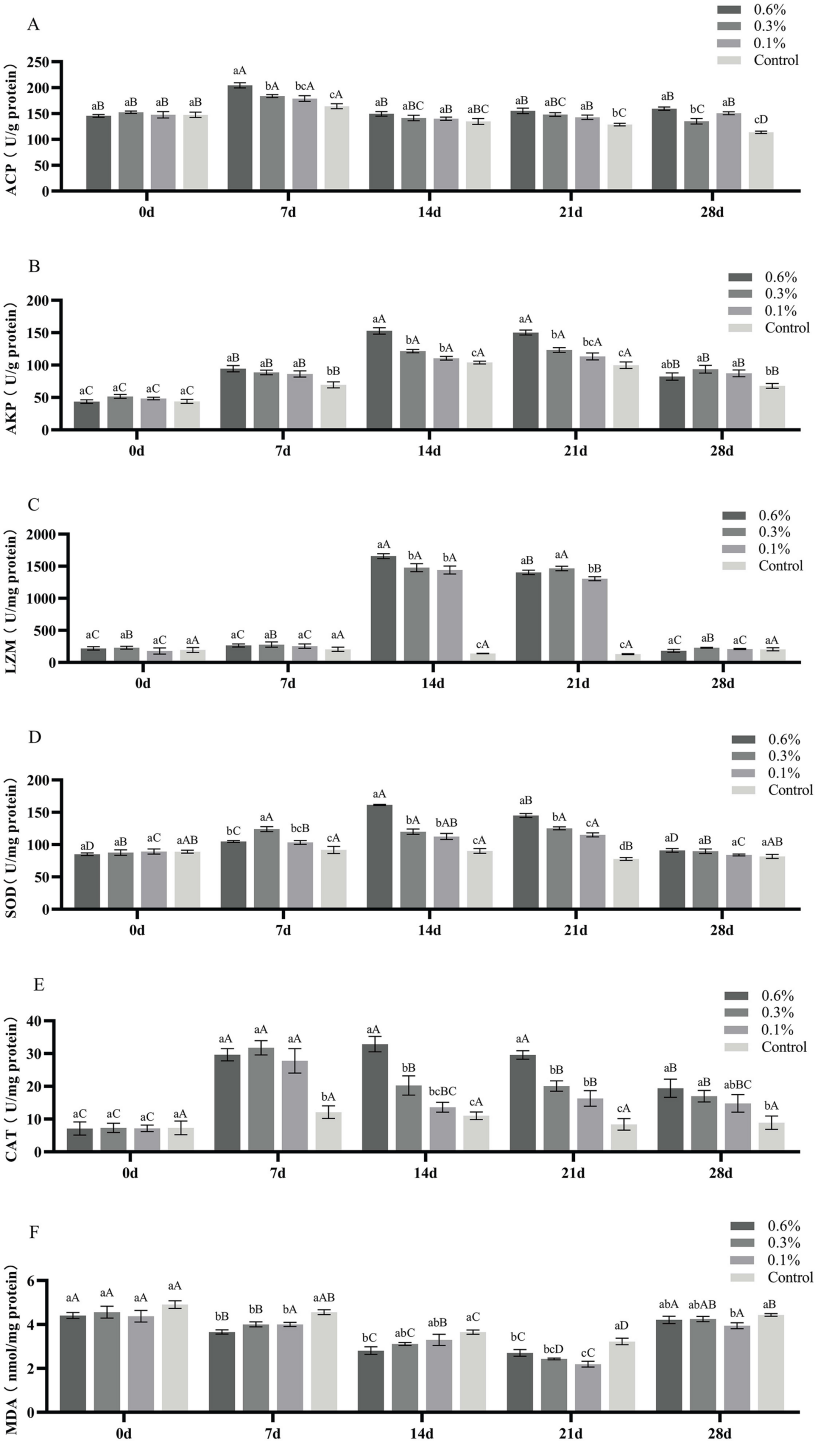
Effect of GF-7 added to the diet on immune enzymes in the liver of *M. salmoides*. A, the ACP activity; B, the AKP activity; C, the LZM activity; D, the SOD activity; E, the CAT activity; F, the MDA content. Data were shown as Mean ± SE (*n* = 9). Different uppercases indicate significant difference (*p* < 0.05) at different time point of the same group. Different lowercases indicate significant difference (*p* < 0.05) among different groups at the same time.

The activities of ACP, AKP, LZM, SOD, CAT and the content of MDA in the liver of *M. salmoides* fed with 0, 0.1, 0.3, and 0.6% GF-7 for 28 days were determined. As shown in Figure 4A, the ACP activity in each administration group was significantly higher than that of the control group on the 7th, 21st, and 28th day (*p* < 0.05), but there was no statistical difference between 0.1, 0.3, 0.6% groups and the control group at the 14th day (*p* > 0.05). The AKP and CAT activity of each dosing group was significantly higher than that of the control group from the 7th to 28th day (*p* < 0.05) (Figures 4B,E). It can be seen from Figure 4C that the activity of LZM in 0.1, 0.3 and 0.6% groups was significantly higher than that of the control group on the 14th and 21st day (*p* < 0.05), which was significantly higher than that of the 7th and 28th day (*p* < 0.05). Compared with the control group, the SOD activity in the three treatment groups increased significantly and the MDA content of each dosing group decreased significantly on the 7th, 14th, and 21st day (*p* < 0.05) (Figures 4D,F).

### 3.5. Effects of GF-7 on immune-related genes in the liver of *Micropterus salmoides*

**Figure 5:**
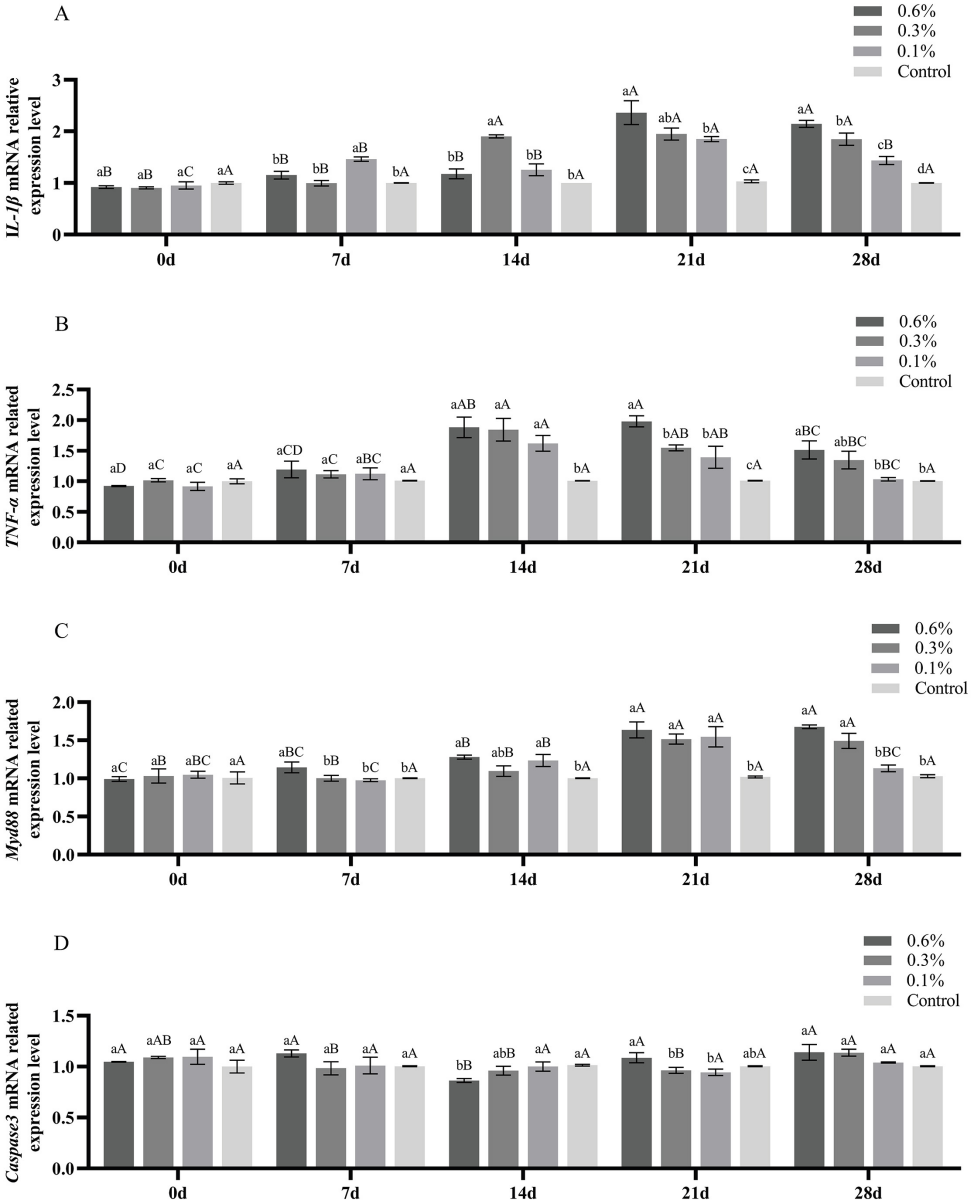
Effect of GF-7 administration on the expression of immune-related genes of *M. salmoides*. A, the IL-1β mRNA related expression level; B, the TNF-α mRNA related expression level; C, the Myd88 mRNA related expression level; D, the Caspase3 mRNA related expression level. Data were shown as Mean ± SE (*n* = 9). Different uppercases indicate significant difference (*p* < 0.05) at different time point of the same group. Different lowercases indicate significant difference (*p* < 0.05) among different groups at the same time.

The gene expression levels of *IL-1β*, *TNF-α*, *Myd88*, and *Caspase3* in the liver of *M. salmoides* fed with GF-7 are shown in Figure 5. On the 21st and 28th day, the expression of *IL-1β* in each treatment group was significantly higher than that in the control group (*p* < 0.05) (Figure 5A). Compared with the control group, the expression of *TNF-α* and *Myd88* in each treatment group was increased significantly at the 14th, 21st, and 28th day (*p* < 0.05) (Figures 5B,C). As shown in Figure 5D, there was no significant difference in the expression of *Caspase3* between each treatment group and the control group (*p* < 0.05).

### 3.6. Bacterial challenge

The challenge test with *A. hydrophila* TPS-30 was conducted after feeding GF-7 for 28 days, and the cumulative mortality and protection were investigated over a week. As shown in Table 3, the protective rates of 0.1, 0.3, and 0.6% groups were 19.4, 30.6, and 47.2%, respectively. The control group showed a 100% mortality rate after the challenge with TPS-30. The cumulative mortality rate in each treatment group was lower than that of the control group, and there was a significant difference between the experimental groups groups (*p* < 0.05).

**Table 3:** Mortality and protection rate of *M. salmoides* with *A. hydrophila* TPS-30 infection for 7 days.

Groups	Mortality rate (%)	Protection rate (%)
0.1% group	80.5 ± 2.8^b^	19.4 ± 2.8^c^
0.3% group	69.5 ± 2.8^c^	30.6 ± 2.8^b^
0.6% group	52.8 ± 2.8^d^	47.2 ± 2.8^a^
Control group	100 ± 0^a^	

Data were shown as Mean ± SE (*n* = 3). Different lowercases indicate significant difference (*p* < 0.05) among different groups.

**Figure 6:**
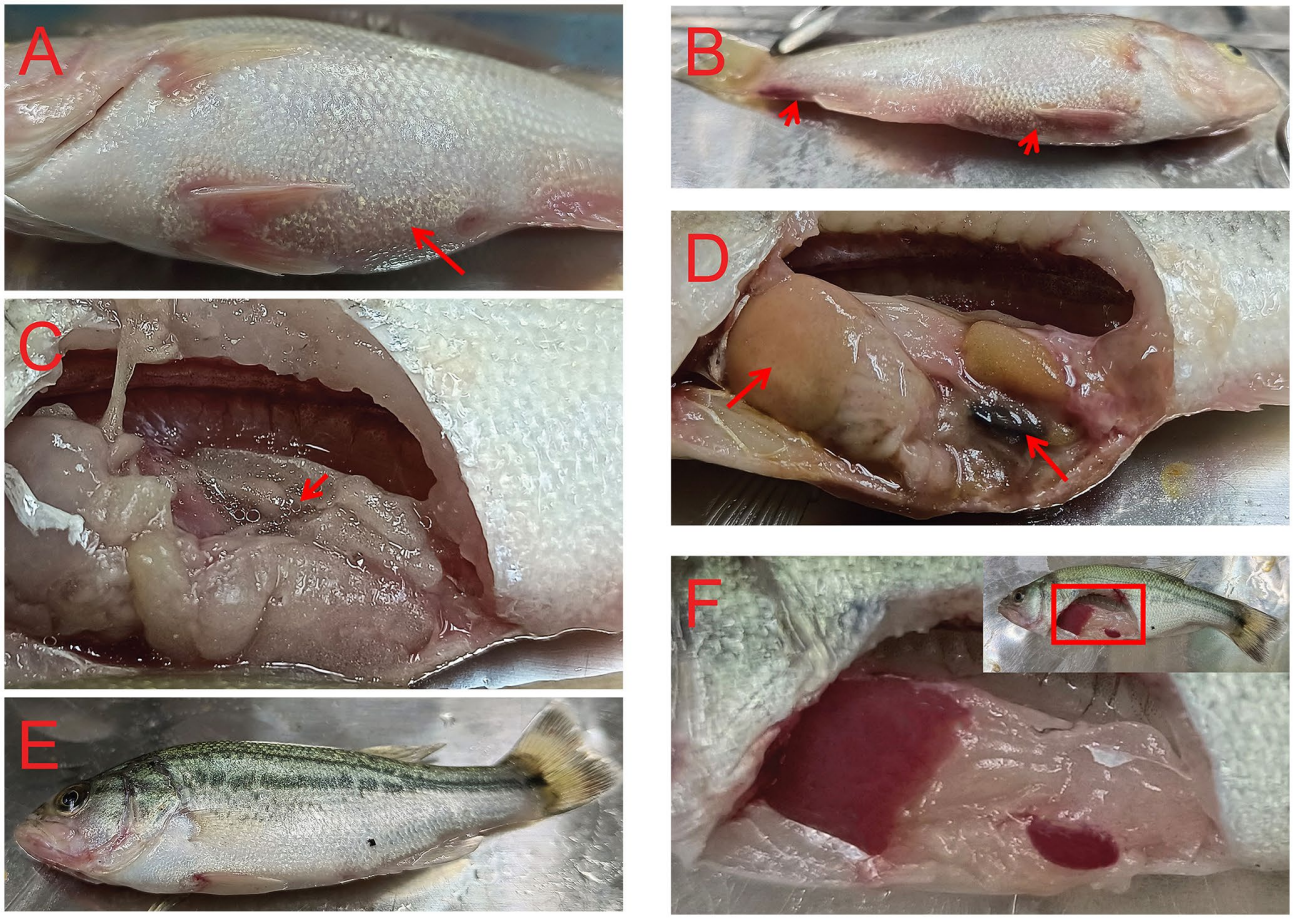
Symptoms of fish infected with *A. hydrophila* TPS-30. **(A–D)** were the symptoms of *M. salmoides* after TPS-30 infection, **(E,F)** were the normal fish, and the arrows in **(A–D)** represented the abdominal distension, body surface bleeding, ascites, the white liver, and the black spleen, respectively.

After the TPS-30 infection, *M. salmoides* moved slowly and reduced food intake. A large number of fish died continuously during the first 3 days, afterwards, there was no fish death. TPS-30 infection could cause bleeding on the body surface and dilate the abdomen. It was found that reddish or yellowish ascites in the abdominal cavity, white liver and black spleen after TPS-30 infection (Figure 6). These symptoms were the same as typical clinical signs of *A. hydrophila* infections (Yuan et al., 2021; Jin et al., 2023).

### 3.7. Liver histopathological analysis

**Figure 7:**
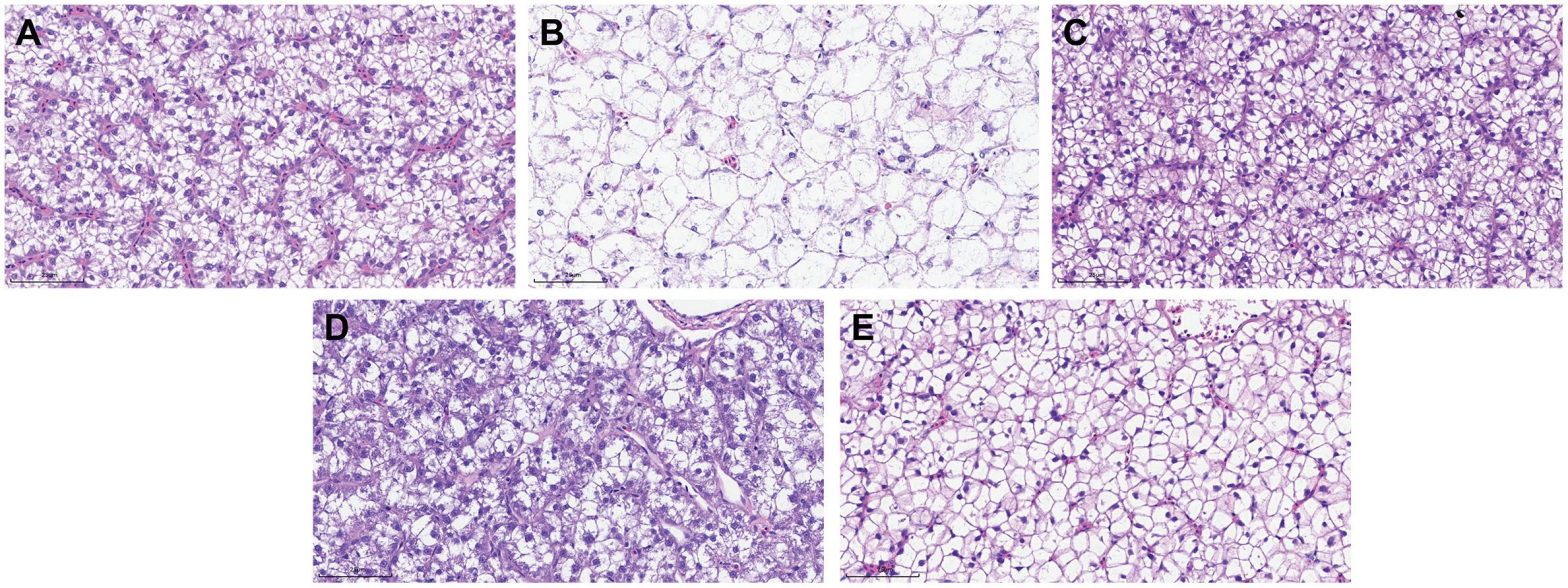
Histopathological changes in the liver of *M. salmoides* (Scale bars, 25 um, 40×). **(A)** Normal group without *A. hydrophila* infection; **(B)** model of *A. hydrophila* infection; **(C)** 0.6% group of *A. hydrophila* infection; **(D)** 0.3% group of *A. hydrophila* infection; **(E)** 0.1% group of *A. hydrophila* infection.

The histological changes of liver tissue structure and cell morphology were observed by H&E staining. As shown in Figure 7A, the normal liver has a complete structure, normal hepatocyte morphology and obvious structure of hepatic sinusoids, and the hepatocytes were arranged in a regular pattern. However, the hepatocytes in the model of *A. hydrophila* infection were seriously vacuolated and swollen, morphology and structure were fuzzy, and the arrangement was disordered, with no obvious hepatic sinusoids (Figure 7B). Surprisingly, the structure and morphology of hepatocytes in the 0.6 and 0.3% groups were improved (Figures 7C,D). While in the 0.1% group, there was slight vacuolation and swelling of hepatocytes (Figure 7E).

## 4. Discussion

Chinese herbal medicines have been used extensively in the aquaculture industry due to their low toxicities and take fewer disadvantages in survival environments (Doan et al., 2020). Quality control of Chinese herbal medicine plays a crucial role in clinical efficacy (Xiong et al., 2018). The types and contents of the active components in Chinese herbal medicines are quite variable, leading to significant variations in the therapeutic benefits, due to the variances in origin, harvesting season, and processing processes. Therefore, it is important to identify the bioactive ingredients of Chinese herbal medicines to carry out quality control. In this study, we used microporous resin to purify the extracts of PoP and SB. We identified the bioactive chemicals including tannins and total flavonoids as the foundation for their quality control. We tested the antibacterial activity of any section of the GC and MS extracts separated by microporous resin and figured out that their bioactive activities were no better than that of their crude extracts. Thus, we combined their extracts to form a special mixture to reduce preparation costs. In addition, the HPLC analysis of the GF-7 fingerprint had been established for the quality control and stability of the efficacy of GF-7.

GF-7 showed a strong inhibitory effect on 7 aquatic pathogens, with MIC values between 0.045 and 0.36 mg/mL, and also reflected a relatively broad antibacterial spectrum. Some studies showed that the MIC values of water extract of GC and SB against *Vibrio parahemolyticus* were 0.49 mg/mL (Yuan et al., 2022) and 3.12 mg/mL (Wu et al., 2021), respectively. Similarly, the MIC values of water extract of GC and SB against *E. trada* were 16 mg/mL when used alone, while the MIC of their combined use was less than 1 mg/mL (Bai et al., 2009). In this study, the MIC of GF-7 against two strains of *E. tarda* were 0.18 and 0.36 mg/mL, respectively, which showed a better antibacterial effect than a single drug, indicating that the combination of Chinese herbal medicines has a synergistic effect. Stratev and Odeyemi (2016) had reviewed the differences in drug resistance among *A. hydrophila* from different food sources. *A. hydrophila* has many serotypes, and it also produces many virulence factors, such as hemolysins, aerolysins, adhesins, enterotoxins, phospholipase and lipase, which may be the reason why strains from different sources have different sensitivities to the same drug. The antibacterial results in this research also showed the effects of drugs can be influenced by the sources of the pathogen strains due to their variation of drug sensitivities. Therefore, the drug sensitivity test of the pathogenic bacteria should be performed to screen drugs in actual use.

As important lysosomal enzymes, ACP and AKP can hydrolyze bacterial lipopolysaccharide and plays a role in killing and digesting microbial pathogens during immune responses (Fu et al., 2017; Bu et al., 2019; Liu et al., 2020). LZM is an important defense molecule of the fish’s innate immune system, which can break the β-1,4 glycosidic bonds between N-acetylmuramic acid and N-acetylglucosamine in bacterial cell wall peptidoglycan and prevent pathogenic bacterial infection (Saurabh and Sahoo, 2008; Bu et al., 2019). The increase of ACP, AKP and LZM activities indicates the improvement of body’s innate immunity. In our research, the upregulation of AKP and LZM activities in each dosing group indicates that GF-7 supplementation might improve the innate immune defense ability of *M. salmoides*. Additionally, we found that SOD and CAT activities were significantly increased after fish were treated with GF-7. The activities of SOD and CAT are directly related to the antioxidant capacity of animals (Xue et al., 2022), which can decompose superoxide radicals into H_2_O and O_2_, and completely remove the excess free radicals in fish (Wang et al., 2022). MDA is a chemical marker of oxidative damage and indirectly reflects lipid peroxidation and cellular damage (Xue et al., 2022). The increase of SOD, CAT activities and the decrease of MDA content indicate that GF-7 is beneficial to protect the liver tissue of *M. salmoides* by antioxidative effects. Our research showed that the trend of ACP, AKP, LZM, SOD, and CAT activity increased and then decreased during the 28 days of feeding GF-7. We speculate that the reason may be that the enzyme activity of *M. salmoides* induced by GF-7 mainly sensitized in the 14–21 days after feeding, which indicates that the optimum time for feeding GF-7 may be 14–21 days.

Inflammation has a potential role in the innate immunity against bacterial challenges. *IL- 1β* and *TNF-α* are the pivotal immune-related pro-inflammatory cytokines in fish, which can be triggered in response to bacterial and viral invasion (Kong et al., 2019; Ibrahim et al., 2021), which can heighten various cellular responses in fish, such as phagocytosis, chemotaxis, macrophage proliferation and lysozyme synthesis (Hou et al., 2022). *Myd88* is a key and universal downstream adapter for most toll-like receptors and plays an important role in both precursors and adaptive immune responses (Gao et al., 2017). The expression level of inflammatory cytokines including *IL-1β* and *TNF-α* in the liver of *M. salmoides* fed with GF-7 was significantly higher than in the control group. Meanwhile, GF-7 could improve the expression of *Myd88* in the liver of *M. salmoides* to a certain extent. The upregulation of the expression level of *IL-1β*, *TNF-α*, and *Myd88* indicates that GF-7 supplementation might induce an immune response in *M. salmoides*, and enhance the body’s resistance to foreign infection (Wang et al., 2022). Therefore, GF-7 could significantly increase the activities of immune-related enzymes and up-regulated the hepatic expression of immune regulators including *IL-1β*, *TNF-α* and *Myd88* of *M. salmoides*, especially on the 14th and 21st days, indicate that GF-7 could improve disease resistance by improving the antioxidant capacity of the body and stimulating autoimmune response.

*A. hydrophila* widely exists in aquatic products and aquaculture environments, which is the main pathogenic bacteria that cause bacterial septicemia and inflammation in fish (Zhang et al., 2020; Dong et al., 2021). Our results showed that GF-7 could significantly reduce the mortality rate of *M. salmoides* infected *A. hydrophila* TPS-30, and its protection rate showed a dose-dependent relationship. At the same time, the histopathological analysis showed that the liver cells of *M. salmoides* attacked by *A. hydrophila* TPS-30 were seriously vacuolated and swollen, while the symptoms of GF-7 treatment group were obviously improved, which further verified that GF-7 could protect *M. salmoides* from *A. hydrophila* infection. However, GF-7 is a Chinese herbal formula with synergistic effects of many components, making it difficult to clarify its mechanism of action, and the specific active ingredients is not yet clear. In the future, we will search for the specific active ingredients responsible for the anti-*A. hydrophila* activity, and to further study their mechanisms of action.

## 5. Conclusion

In conclusion, GF-7 had excellent antibacterial activity against various aquatic pathogenic bacteria *in vitro*. Meanwhile, GF-7 could significantly enhance immune-related enzyme activity and gene expression of *M. salmoides*, and had a good protective effect on *M. salmoides* infected with *A. hydrophila*. These findings imply that GF-7 is a potential natural medicine for the prevention and treatment of numerous aquatic pathogenic infectious diseases and is worth further application for aquaculture.

## Data availability statement

The original contributions presented in the study are included in the article/Supplementary material, further inquiries can be directed to the corresponding authors.

## Ethics statement

The animal study was reviewed and approved by Institutional Animal Care and Use Committee of Zhejiang Institute of Freshwater Fisheries.

## Author contributions

HaG and JY designed the experiments and revised the article. HuG completed all experiments and wrote the article. JC performed data analysis and wrote the article. XY, JZ, and JW helped to complete some experiments. All authors contributed to the article and approved the submitted version.

## Funding

This work was supported by Zhejiang Provincial Public Welfare Technology Application Research (Grant No: LGN22C190007), Key Research and Development Programs of Huzhou City (Grant No: 2019ZD2027), and Key Research and Development Programs of Zhejiang Province (Grant No: 2022C02027).

## Conflict of interest

The authors declare that the research was conducted in the absence of any commercial or financial relationships that could be construed as a potential conflict of interest.

## Publisher’s note

All claims expressed in this article are solely those of the authors and do not necessarily represent those of their affiliated organizations, or those of the publisher, the editors and the reviewers. Any product that may be evaluated in this article, or claim that may be made by its manufacturer, is not guaranteed or endorsed by the publisher.
