# Supplementary material for: The combined effect of a novel formula of herbal extracts on bacterial infection and immune response in Micropterus salmoides
Source: Front Microbiol. 2023 Jun 2;14:1185234. doi: 10.3389/fmicb.2023.1185234 (PMC10272801; doi:10.3389/fmicb.2023.1185234)
Supplement: Supplementary file 1 [file Data_Sheet_1.docx]

***Supplementary Material***

**The combined effect of a novel formula of herbal extracts on bacterial infection and immune response in** ***Micropterus salmoides***

**Huanyu Guo^1^, Jing Chen^2^, Xuemei Yuan^2^, Jian Zhang^3^, Jiayang Wang^1^, Jiayun Yao^2*^ and Haixia Ge^1*^**

^1^ College of Life Sciences, Huzhou University, Huzhou, Zhejiang, China

^2^ Key Laboratory of Healthy Freshwater Aquaculture, Ministry of Agriculture and Rural Affairs, Key Laboratory of Fish Health and Nutrition of Zhejiang Province, Zhejiang Institute of Freshwater Fisheries, Huzhou, Zhejiang, China

^3^ State Key Laboratory of Natural Medicines, China Pharmaceutical University, Nanjing, China

***** **Correspondence:** Haixia Ge: [ghxzwx2012@163.com](mailto:ghxzwx2012@163.com); Jiayun Yao: yaojiayun@126.com.

**Preparations and quantitative analysis of novel combined prescription GF-7 of herbal extracts**

1. **Preparation of *Galla Chinensis* (GC) extract**

We used “*dubei*” with a better quality to prepare GC extract (Ren et al., 2021). The dried GC (1 kg) was crushed into powders, and added 15L 50% ethanol to soak for 2 hours, then heated and refluxed for 2 hours. After extraction, the GC was filtered, and the plant residues was extracted and filtered again. The supermentant were concentrated to remove the solvent, then dried at 60℃ to obtain the GC extract. The tannin content in GC extract was determined according to the method mentioned in general rule 2202 of the Chinese Pharmacopoeia (2020).

The extraction rate of GC was 65% and the level of tannins in the GC extract was 72.0%.

1. **Preparation of *Mangosteen Shell* (MS) extract**

MS was obtained by removing the pulp of the purchased fresh *mangosteen* fruit and dried in an oven at 60°C. The dried MS (1 kg) was crushed into powders and added 15 times of 70% ethanol to soak for 2 hours, then heated and refluxed for 2 hours. After extraction, the MS was filtered, and the plant residues was extracted and filtered again. The supermentant were concentrated to remove the solvent, then dried at 60°C to obtain the dry MS extract. The α-mangostin as one of the major active constituents in the MS (Wittenauer et al., 2012) was determined by HPLC. The HPLC method is shown as follows: 0.5% formic acid in water (eluent A, 87%) and methanol (eluent B, 13%) at the flow rate of 1.0 mL/min and DAD detection at 254 nm.

The extraction rate of MS was 17%, and α-mangostin and γ-mangostin are the major active ingredients in MS and can be used as the indicator components for quality control (Aizat et al., 2019). As shown in **Figure S1**, the percentages of α-mangostin and γ-mangostin in the MS are 20.5% and 4.95%, respectively.


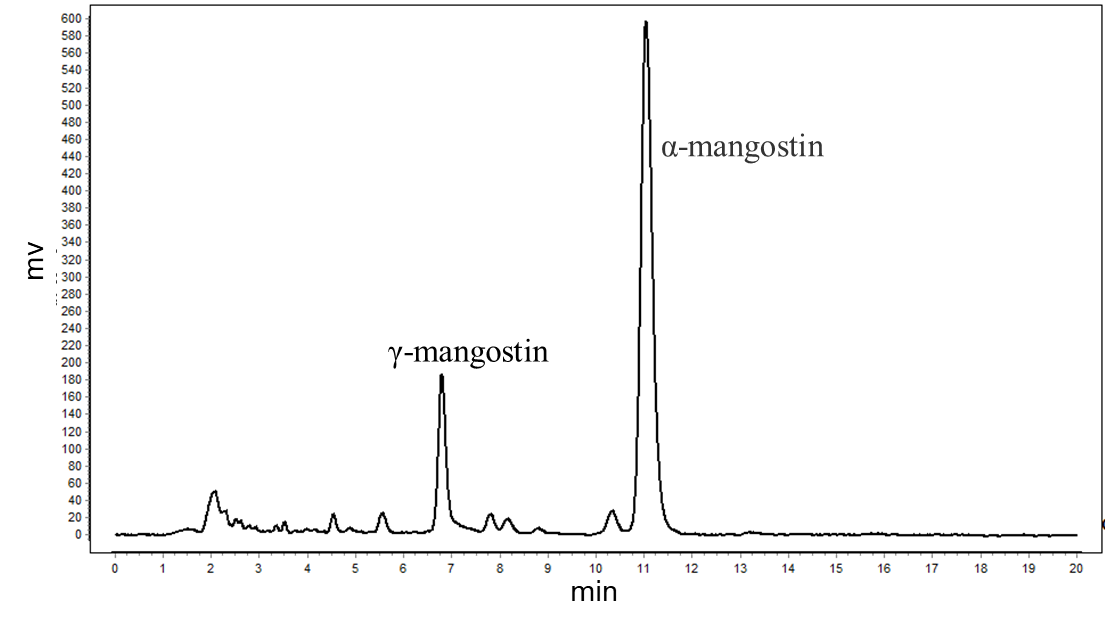


**Figure S1** The HPLC chromatogram of the MS extract. Eight μL of 1.01 mg/mL of MS extract was subjected to HPLC-DAD analysis, and the chemical fingerprint was revealed at the wavelength 254 nm. By comparing the standards, it is identified that the main peaks are γ-mangostin and α-mangostin.

1. **Preparation of** ***Pomegranate peel* (PoP) extract**

PoP is the peel of *Punica granatum* L., and the dried PoP (1 kg) was crushed into powders. 15 times of 50% ethanol was added to soak for 2 hours, then heated and refluxed for 2 hours. After extraction, the PoP was filtered, and the plant residues was extracted and filtered again. The supermentant were concentrated to remove the solvent, then dried at 60℃ to obtain the dry PoP extract. Next, the PoP extract was dissolved in water by ultrasound, and slowly adsorbed on DM301 macroporous resin for overnight, then was eluted with water, 50% ethanol, and 95% ethanol in gradient to obtain different eluting fractions. The tannin content in each eluting fractions was determined by the method mentioned in general rule 2202 of the Chinese Pharmacopoeia (2020).

The extraction rate of PoP was 41%. We observed that 50% alcohol eluting fraction of the PoP extract shows the significantly effective antibacterial activity. Tannins in PoP extract play an important role in its biological activity (Mo et al., 2022). The tannin content was increased from 22.6% of the crude extract to 49.2% of the effective section, and its weight was 37.1% of the extract. The water eluting fraction and 95% alcohol eluting fraction contained less than 10% of tannin, and both of these fractions show no obvious antibacterial activity. Therefore, the 50% alcohol eluting fraction of PoP was selected as the effective fraction involved the formula of GF-7.

1. **Preparation of *Scutellaria baicalensis* (SB) extract**

The dried SB (1 kg) was crushed into powders and added 15 times of 70% ethanol to soak for 2 hours. After extraction, the PoP was filtered, and the plant residues was extracted and filtered again. The supermentant were concentrated to remove the solvent, then dried at 60℃ to obtain the dry SB extract. Next, the SB extract was dissolved in water by ultrasound, slowly adsorbed on HPD100 macroporous resin for overnight, and was eluted with water, 70% ethanol, and 95% ethanol in gradient to obtain different eluting fractions. The content of total flavonoids in each eluting fraction was determined by UV spectrophotometry according to Ren et al. (Ren et al., 2017) with some modifications.

The extraction rate of SB was 33%. The 70% alcohol eluting fraction from SB has been observed to show significantly enhanced antibacterial activity; Flavonoids in SB are the main active substances (Zhao et al., 2019) and the content of total flavonoids was increased from 43.8% of the crude extract to 75.2% of the effective fraction, and its weight was 50% of the extract. The water eluting fraction and 95% alcohol eluting fraction with 7.9% and 7.0% of total flavonoids, respectively, shows no significant antibacterial activity. Therefore, the 70% alcohol eluting fraction of SB was selected as the effective part involved in the GF-7.

1. **Preparation of GF-7**

The novel combination GF-7 was composed of *Galla Chinensis* (GC) extract, *Mangosteen Shell* (MS) extract, effective fraction of *Pomegranate peel* (PoP), and effective fraction of *Scutellaria baicalensis* (SB) in equal proportions.

**The simple flow chat of the experimental design**


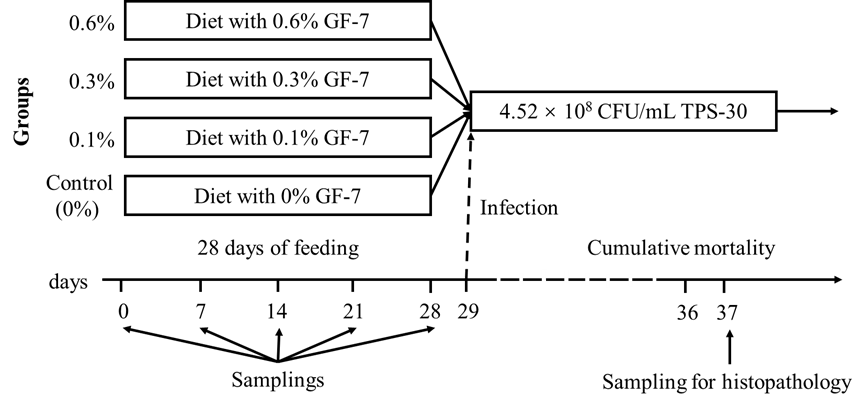


**Figure S2.** The experimental design of protective effect of GF-7 on *Micropterus salmoides* infected with *Aeromonas hydrophila*. GF-7 was continuously fed for 28 days, and samples were taken at the 0th, 7th, 14th, 21st and 28th day, and 0.1 mL TPS-30 was injected intraperitoneally into the fish at the 29th day.

**References**

Aizat, W.M., Ahmad-Hashim, F.H., and Jaafar, S.N.S. (2019). Valorization of mangosteen, "The Queen of Fruits," and new advances in postharvest and in food and engineering applications: A review. *Journal of Advanced Research* 20**,** 61-70. doi: 10.1016/j.jare.2019.05.005.

Mo, Y., Ma, J., Gao, W., Zhang, L., Li, J., Li, J., et al. (2022). Pomegranate Peel as a Source of Bioactive Compounds: A Mini Review on Their Physiological Functions. *Frontiers in Nutrition* 9**,** 887113. doi: 10.3389/fnut.2022.887113.

Ren, Q., Xue, Y., Nie, Q., Wang, Y., and Xia, T. (2017). Determination of total flavonoids from different production areas of Scutellaria barbata D. Don by UV Spectrophotometry. *Journal of Jining Medical Univisity* 40(2)**,** 103-105. doi: 10.3969/j.issn.1000-9760.2017.02.006.

Ren, Y., Zhang, X., Li, T., Zeng, Y., Wang, J., and Huang, Q. (2021). Galla Chinensis, a Traditional Chinese Medicine: Comprehensive review of botany, traditional uses, chemical composition, pharmacology and toxicology. *Journal of Ethnopharmacology* 278**,** 114247. doi: 10.1016/j.jep.2021.114247.

Wittenauer, J., Falk, S., Schweiggert-Weisz, U., and Carle, R. (2012). Characterisation and quantification of xanthones from the aril and pericarp of mangosteens (Garcinia mangostana L.) and a mangosteen containing functional beverage by HPLC–DAD–MSn. *Food Chemistry* 134(1)**,** 445-452. doi: 10.1016/j.foodchem.2012.02.094.

Zhao, T., Tang, H., Xie, L., Zheng, Y., Ma, Z., Sun, Q., et al. (2019). Scutellaria baicalensis Georgi. (Lamiaceae): a review of its traditional uses, botany, phytochemistry, pharmacology and toxicology. *Journal of Pharmacy and Pharmacology* 71(9)**,** 1353-1369. doi: 10.1111/jphp.13129.
